# Decrease in *Mycobacterium ulcerans* disease (Buruli ulcer) in the Lalo District of Bénin (West Africa)

**DOI:** 10.1186/s12879-019-3845-2

**Published:** 2019-03-12

**Authors:** Esaï Gimatal Anagonou, Roch Christian Johnson, Yves Thierry Barogui, Ghislain Emmanuel Sopoh, Gilbert Adjimon Ayelo, Akpeedje Carolle Wadagni, Jean Gabin Houezo, Didier Codjo Agossadou, Michel Boko

**Affiliations:** 1 Centre Inter-Facultaire de Recherche en Environnement pour le Développement Durable, Université d’Abomey-Calavi, Abomey-Calavi, Bénin; 2 Programme National de Lutte contre la Lèpre et l’Ulcère de Buruli, Cotonou, Bénin; 3 Centre de Dépistage et de Traitement de l’Ulcère de Buruli de Lalo, Lalo, Bénin; 4 Centre de Dépistage et de Traitement de l’Ulcère de Buruli d’Allada, Allada, Bénin; 5 Institut Régional de Santé Publique, Ouidah, Bénin

**Keywords:** Buruli ulcer, *Mycobacterium ulcerans*, Prevalence rate, Decrease, Neglected tropical disease

## Abstract

**Background:**

Buruli ulcer (BU) is a chronic, necrotizing infectious skin disease caused by *Mycobacterium ulcerans*. In recent years, there has been a decrease in the number of new cases detected. This study aimed to show the evolution of its distribution in the Lalo District in Bénin from 2006 to 2017.

**Methods:**

The database of the BU Detection and Treatment Center of Lalo allowed us to identify 1017 new cases in the Lalo District from 2006 to 2017. The annual prevalence was calculated with subdistricts and villages. The trends of the demographic variables and those related to the clinical and treatment features were analysed using Microsoft Excel® 2007 and Epi Info® 7. Arc View version® 3.4 was used for mapping.

**Results:**

From 2006 to 2017, the case prevalence of BU in the Lalo District decreased by 95%. The spatial distribution of BU cases confirmed the foci of the distribution, as described in the literature. The most endemic subdistricts were Ahomadégbé, Adoukandji, Gnizounmè and Tchito, with a cumulative prevalence of 315, 225, 215 and 213 cases per 10,000 inhabitants, respectively. The least endemic subdistricts were Zalli, Banigbé, Lalo-Centre and Lokogba, with 16, 16, 10, and 5 cases per 10,000 inhabitants, respectively. A significant decrease in the number of patients with ulcerative lesions (*p* = 0.002), as well as those with category 3 lesions (*p* < 0.001) and those treated surgically (p < 0.001), was observed. The patients confirmed by PCR increased (from 40.42% in 2006 to 84.62% in 2017), and joint limitation decreased (from 13.41% in 2006 to 0.0% in 2017).

**Conclusion:**

This study confirmed the general decrease in BU prevalence rates in Lalo District at the subdistrict and village levels, as also observed at the country level. This decrease is a result of the success of the BU control strategies implemented in Bénin, especially in the Lalo District.

## Background

Buruli ulcer (BU) is a chronic, necrotizing infectious skin disease caused by *Mycobacterium ulcerans.* BU is the most widespread mycobacterial disease worldwide after tuberculosis and leprosy [1]. Symptoms usually starts with nodules, plaques or oedema, and these lesions can evolve into massive skin ulcerations when detected late or left untreated. The bone may be affected in some cases. The disease usually affects children in tropical and subtropical areas where endemic foci are almost always organized around aquatic ecosystems [2]. BU diagnosis is based on clinical and epidemiological features defined by the World Health Organization (WHO) [3]. Four laboratory tests are currently available for BU diagnosis confirmation [4]. Among these tests, the two most commonly used are a direct smear examination (DSE) to detect acid-fast bacilli (AFB) and *IS2404* PCR, which is the most sensitive test to date [4–6].

The mode of transmission of this disease is not completely elucidated, but several hypotheses have been proposed. Exactly how *M. ulcerans* is introduced into the skin of humans remains unknown; however, unlike tuberculosis or leprosy, the infection is acquired directly or indirectly from the environment and not from contact with other patients [7].

The management of BU requires the use of antibiotics (streptomycin or clarithromycin associated with rifampicin) taken daily for 8 weeks. In addition to antibiotics, wound care is an important component of treatment. In the most severe cases, surgery may be required.

BU was recognized for the first time in 1897 in Buruli County in Uganda by Sir Albert Cook. MacCallum was the first to publish the description of the disease in Australia [8]. In 1961, a large case series of the disease was described in Buruli, now Nakasongola, District in Uganda [9]. The disease was subsequently reported in more than 30 countries, mostly in tropical and subtropical areas, but the prevalence in many endemic countries in West Africa remains uncertain [10].

Africa appears most affected. However, some outbreaks were reported in Australia, French Guiana, Peru, Papua New Guinea and Japan [11–17].

In Australia, sporadic cases have been observed in the State of Victoria in the Bairnsdale area since the 1930s [6]. Most new cases are associated with the central coastal area of ​​the State of Victoria.

In Bénin, the observations made in the districts of Lalo [18] and Zè [19] in southern Bénin showed a focal distribution of BU in endemic areas. Thus, the detection of the disease can vary from one subdistrict to another within the same district and from one village to another within the same subdistrict. It is also observed that the number of new cases detected yearly has decreased since 2007 [20]. Furthermore, some variations can be noticed according to some clinical or epidemiological characteristics of patients. Several studies have been performed to determine the epidemiology and distribution of BU disease [18, 19], but no study has described its features over time and space. The objective of this work was to analyse changes in BU epidemiological and clinical features over time and space from 2006 to 2017 in Lalo, one of the most endemic districts in Bénin, and to discuss possible contributing factors.

## Methods

### Study area

The study was carried out in the Lalo District in Bénin. Lalo is one of the six administrative subdivisions of the Couffo Department and covers an area of 432 km^2^ [21]. The climate is Sudano-Guinean, which is characterized by a low temperature of approximately 27 °C with few variations. This type of climate allows a succession of four (two dry and two rainy) seasons per year, which favours agriculture practices in the district. Like most districts of the Couffo Department, Lalo is located on the Aplahoué clay plateau, with an average altitude of 80 m [21]. The localities to the northeast and east of the district are irrigated by the Couffo River over 11 km and its effluents; there are also some swamps. Lalo District is administratively divided into 11 subdistricts: Lalo-Centre, Lokogba, Gnizounmè, Banigbé, Zalli, Hlassamè, Adoukandji, Ahomadégbé, Ahodjinnako, Tohou and Tchito. The subdistricts are subdivided into 67 villages. Each village consists of several hamlets that aggregate households linked by kinship.

### Study design and population

The retrospective data from 2006 to 2017 used in this study were obtained using the BU02 form from the WHO [22]. We included all cases of BU detected and treated at the CDTUB in the Lalo District from 2006 to 2017. All included cases were clinically validated and treated at the CDTUB of Lalo by a well-trained medical team. Three levels were considered in our study: the district, the subdistrict and the village levels. At the subdistrict level, the four most prevalent subdistricts were considered. The cases were sorted according to the origin of the patient and the year from 2006 to 2017 to show the trend of geographical distribution. The epidemiological, clinical and treatment features of the disease were analysed at the district level.

### Variables

The variables used in this study included demographic data (age and sex of patients), clinical data including clinical forms (non-ulcerated lesions: nodule, plaque, oedema; ulcerated lesions: ulcer, osteomyelitis), WHO categories of lesions (category 1: a single lesion ≤5 cm in diameter; category 2: a single lesion 5–15 cm in diameter; category 3: a single lesion > 15 cm in diameter; multiple lesions; lesions of critical sites such as the face, breast or external genital organs; and osteomyelitis) [3], localizations of the lesions (upper localization: head, arms and trunk; lower locations: buttock, leg, foot), the joint limitation (when case was detected), cases confirmation by PCR, and variables related to the treatment (surgery and no surgery).

### Data processing and analysis

The annual prevalence was calculated by district, subdistrict and village. The cumulative prevalence was calculated using population data that had been updated annually from 2006 to 2017 [23]. The trends of the demographic variables and those related to the clinical and treatment features were analysed using Microsoft Excel® 2007. Epi Info® 7 was used to measure the association between two variables. Arc View® version 3.4 was used for mapping.

## Results

A total of 1017 cases of BU were detected from 2006 to 2017 in the district of Lalo.

### Distribution of BU in the district of Lalo (2006–2017)

Table 1 shows the BU prevalence (cases per 10,000 inhabitants) from 2006 to 2017. There was a striking overall decrease in the prevalence during the study period in the district of Lalo (from 22 in 2006 to 1 in 2017, − 95%). This decrease was also observed in all subdistricts. In Ahomadégbé, the prevalence decreased from 73 in 2006 to 5 in 2017. This decrease was approximately 7 cases per 10,000 inhabitants per year. In Adoukandji, the prevalence decreased from 52 in 2006 to 1 in 2017 (approximately 6 cases per 10,000 inhabitants per year). In Gnizounmè, the prevalence decreased from 42 in 2006 to 4 in 2017 (approximately 5 cases per 10,000 inhabitants per year). In Tchito, the prevalence decreased from 41 in 2006 to 2 in 2017 (approximately 4 cases per 10,000 inhabitants per year).

**Table 1:**
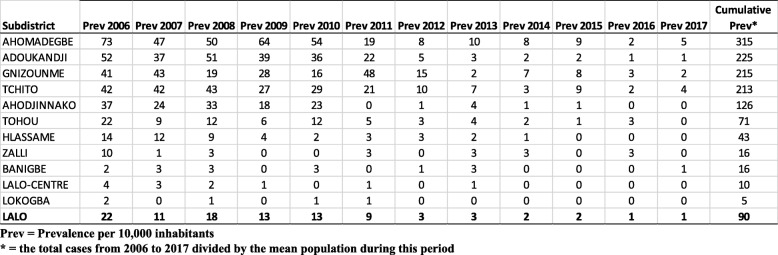
Trend of BU prevalence from 2006 to 2017

### Spatial distribution of BU and the trend over time in the Lalo District and the four most endemic subdistricts

**Fig. 1:**
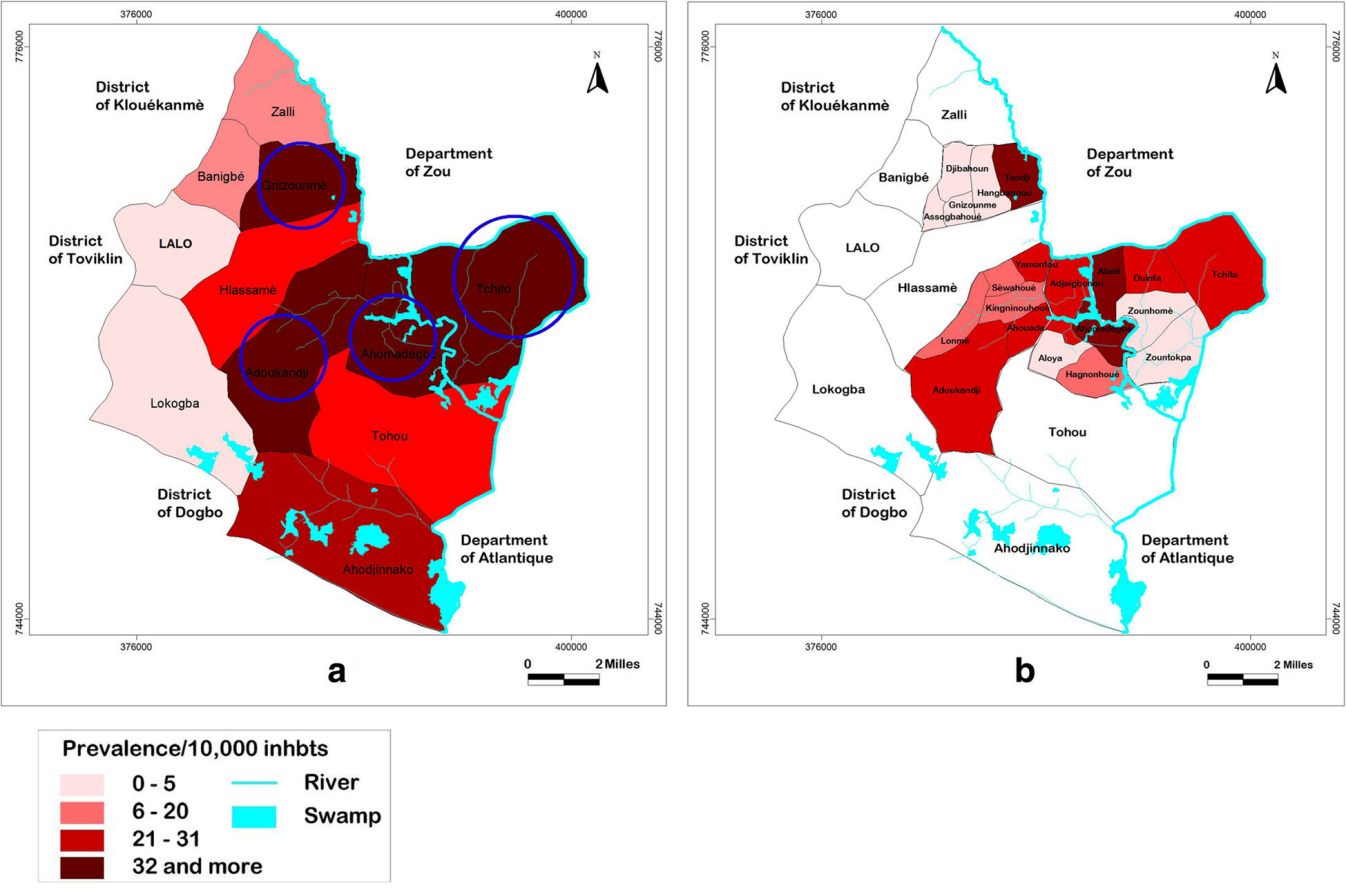
Prevalence of BU in Lalo district (**a**) and the four most endemic subdistricts (**b**)

The subdistricts of Ahomadégbé, Adoukandji, Gnizounmè and Tchito have respectively cumulative prevalence of 315, 225, 215, and 213 cases per 10,000 inhabitants (Table 1). Spatial mapping of BU in the Lalo district and in these four most endemic is presented in Fig. 1. Figure 1a shows the cumulative prevalence at the subdistrict level, and Fig. 1b shows the prevalence at the village level in the four most endemic subdistricts. At both levels, endemicity varied from one subdistrict to another (Fig. 1a) and from one village to another (Fig. 1b). In subdistrict Ahomadégbé, the village with the greatest prevalence was Ahomadégbé-Centre (710 cases per 10,000 inhabitants), and Aloya had the least prevalence (22 cases per 10,000 inhabitants). Other villages, such as Adjaïgbonou and Hagnonhoué, had moderate prevalence rates (342 and 137 cases per 10,000 inhabitants, respectively). Of the four villages, only Aloya has no river or swamp. In the Adoukandji subdistrict, the most endemic villages were Adoukandji-Centre and Yamontou (284 and 266 cases per 10,000 inhabitants, respectively), and the least endemic villages were Sèwahoué and Kingninouhoué (123 and 111 cases per 10,000 inhabitants, respectively). Other villages, such as Lonmè and Ahouada, had moderate prevalence rates (145 and 217 cases per 10,000 inhabitants, respectively). In the Gnizounmè subdistrict, only Tandji village had a high prevalence (1134 cases per 10,000 inhabitants); the others, such as Hangbannou, Djibahoun and Gnizounmè-Centre, had a prevalence varying from 53 to 74 cases per 10,000 inhabitants. In the Tchito subdistrict, the most endemic village was Aboti (645 cases per 10,000 inhabitants), and the least endemic village was Zountokpa (70 cases per 10,000 inhabitants). Other villages, such as Ouinfa and Tchito-Centre, had moderate prevalence rates (229 and 321 cases per 10,000 inhabitants, respectively). In contrast to Aloya village, Sèwahoué, Kingninouhoué, Hangbannou, Zountokpa villages have swamps and rivers.

**Fig. 2:**
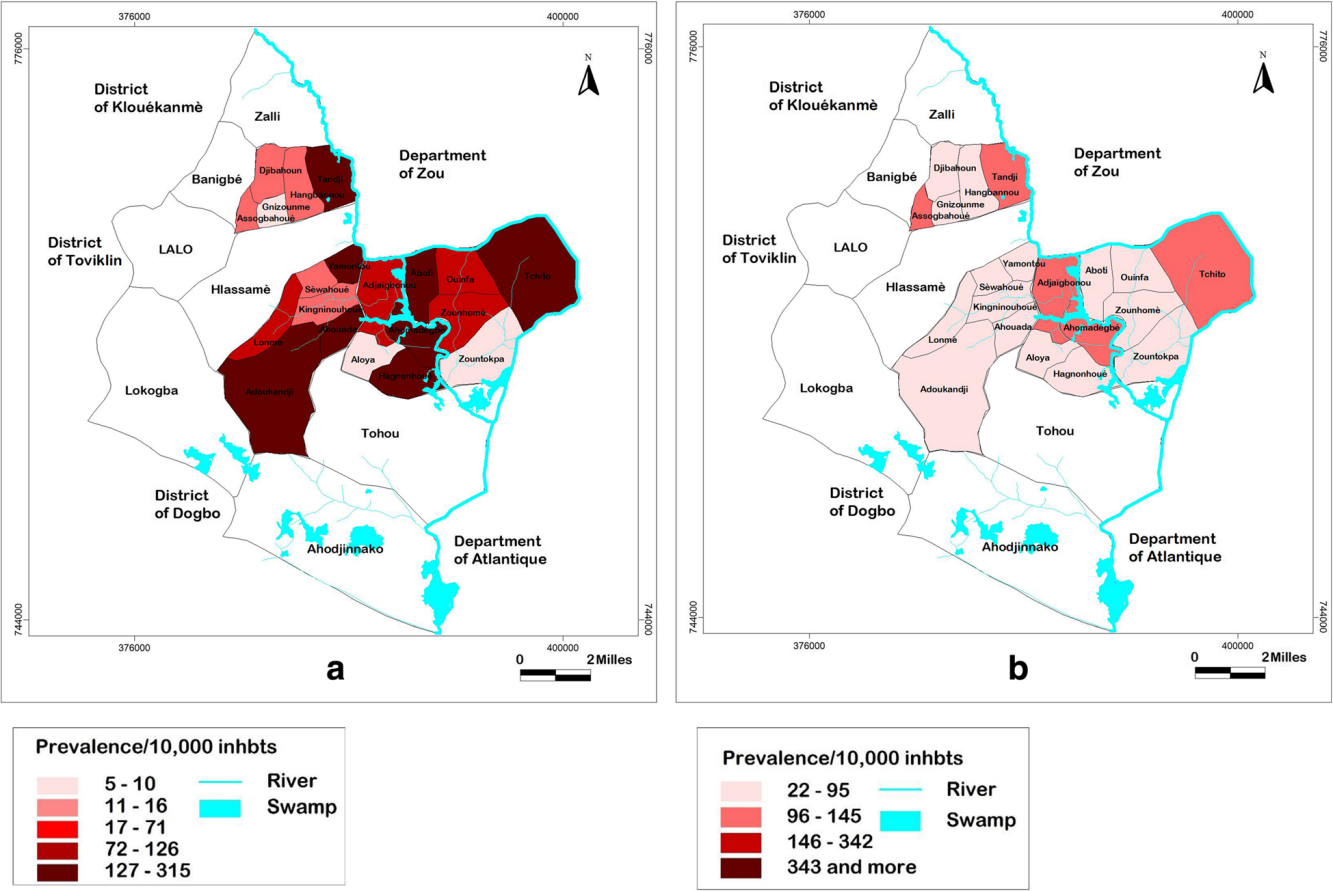
The rivers and swamps are shown with blue colour. The prevalence per 10,000 inhabitants was represented on the maps by range of colours, from bright red to dark red. Lighter is the colour, least endemic the locality is; darker is the colour, more endemic the locality is. **a** Prevalence of BU in the four most endemic subdistricts in 2006, **b** Prevalence of BU in the four most endemic subdistricts in 2017

The map of the trend of BU prevalence in the four most endemic subdistricts of Lalo District is presented in Fig. 2. Figure 2a shows the prevalence in 2006, and Fig. 2a shows the prevalence in 2017. In the subdistrict of Ahomadégbé, the prevalence decreased over time in all villages, but Ahomadégbé-Centre and Adjaïgbonou still had higher prevalence rates than those in other villages in 2017 (Fig. 2b). In the subdistrict of Adoukandji, the disease disappeared completely in 2017 (Fig. 2b). The prevalence decreased in each village of the subdistrict of Gnizounmè, but the villages of Tandji and Assogbahoué remained the most endemic in 2017, while the disease disappeared in all other villages (Fig. 2b). In the subdistrict of Tchito, the disease disappeared in all villages in 2017 except in Tchito-Centre (Fig. 2b).

In the four subdistricts, all villages that presented as none or least endemic had detected cases at some points during the study period.

### Epidemiological, clinical and therapeutic characteristics of patients

The epidemiological, clinical and therapeutic characteristics of the 1017 patients are presented in Table 2. The median (Q1; Q3) age was 12 years (8; 30), ranging from 1 to 92. A total of 330 (32.45%) patients were confirmed by PCR. A total of 560 (55.06%) patients were aged < 15 years, and 491 (48.28%) patients were male. A total of 726 (71.39%) patients had ulcerated lesions, and 586 (58.37%) patients had lesions located on the lower limbs. A total of 436 (43.43%) patients had WHO category 3 lesions, and 89 (8.75%) cases had joint limitations. A total of 538 (53.59%) cases were treated surgically. The trend over time of these epidemiological, clinical and therapeutic indicators showed a significant decrease in ulcerative lesions (from 81.01% in 2006 to 53.85% in 2017; *p* = 0.002, df = 11). Severe lesions (WHO category 3) decreased significantly (from 79.49% in 2006 to 23.08 in 2017; *p* < 0.001, df = 11). Similarly, there was a significant reduction in patients with joint limitation (from 13.41% in 2006 to 0.0% in 2017; *p* < 0.001, df = 22) and patients treated with surgery (from 94.97% surgeries in 2006 to 23.08% in 2017; *p* < 0.001, df = 11). There was a significant increase in patients confirmed by PCR (from 40.42% in 2006 to 84.62% in 2017; *p* < 0.001, df = 22).

**Table 2:**
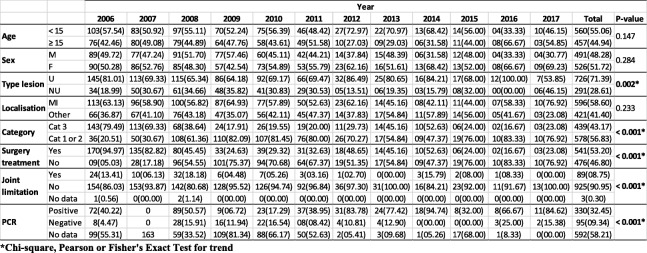
Trends over time of epidemiological, clinical and therapeutic characteristics of patients

## Discussion

This study was conducted in the Lalo District, one of the most endemic districts for BU in Bénin. All 11 subdistricts and 61 villages were included in the study from 2006 to 2017. A total of 1017 cases were reported following the WHO BU diagnosis criteria [3] and were recorded with the WHO BU02 form [22].

Our study, in accordance with the data of the literature [18, 19], confirms the focal character of the distribution of BU. In an endemic district, there are endemic and non-endemic villages. However, in endemic subdistricts, there was not any village that was purely non-endemic throughout the study period, although the endemicity varied from one village to another. This raises the question of a consensual case definition of endemicity for an area. Indeed, one village can be endemic for two or three years, and the following year, the village may no longer have cases despite active case detection efforts. The reason for this inconsistency in the appearance of the disease in an area are unknown.

Several hypotheses could explain this focal distribution. The distribution may have been influenced by the presence of the causative agent in the environment. A previous study conducted in Bénin demonstrated an association between the prevalence of BU and the presence of *M. ulcerans* in the environment (natural water sources, vegetables) [24]. Water bugs and other insects associated with the roots of aquatic plants have been shown to be vectors of *M. ulcerans* [25]. The mycobacteria had been cultivated in an aquatic Hemiptera (water strider, *Gerris* sp.) [26].

Environmental factors alone cannot explain the distribution of BU. It was demonstrated that the distance between a village and a natural water source (swamp or river) and the use of unclean water for domestic activities could play important roles in the frequency of infection with *M. ulcerans* [18]. Thus, human activities related to the aquatic ecosystem may also play important roles. Some studies have shown an association between agricultural practices, specifically rice cultivation, and the prevalence of BU [27, 28]. In our study area, rice cultivation was practised in Ahomadégbé-Centre village. This could explain why the prevalence was higher in Ahomadégbé-Centre than that in the other villages (Fig. 1).

There was an overall downward trend in the detection of BU cases. In 2006, the detection rate was 22 cases per 10,000 inhabitants in the district of Lalo; however, in 2017, it was 1 case per 10,000 inhabitants (Table 1). This decrease was also observed at the subdistrict level (Table 1). Several hypotheses may explain this decrease. First, Bénin has good epidemiological surveillance strategies based on the use of the WHO BU02 form [29]. This resulted in quality data for the study period. Patients who had BU for several years before the establishment of the control system were treated. Once patients have been cured, only the incident cases are detected. In our study, the proportion of patients confirmed by PCR increased significantly (from 40.42% in 2006 to 84.62% in 2017). This potentially means that there was a reduction in the over-diagnosis of BU that may have occurred in earlier years with clinical diagnosis alone. This could generate the observed decrease in new cases, but this is likely not a sufficient reason. Changes in the environment could cause major ecological disturbances, including modification of the habitat of *M. ulcerans*. Therefore, there could be a decline in new cases as a result of the decrease or absence of *M. ulcerans* in the environment. The improvement of drinking water access for populations in the district of Lalo may also reduce human contact with the aquatic ecosystem that could be a risk factor in the transmission of BU. Indeed, according to the literature, the major endemic areas of BU are found mostly in poorly drained swamp regions where access to clean water is limited [18, 30]. In the subdistrict of Gnizounmè, for example, since 2005, access to drinking water has improved, which has reduced human contact with the aquatic ecosystem, thus minimizing the risk of infection with *M. ulcerans*.

The age distribution confirms a predominance of lesions in children (55,18% of all cases < 15 years old), as does the literature [31–33]. This age group, especially children 5–15 years of age or older, is more easily exposed (by walking to do laundry and obtaining water) and susceptible to infection with *M. ulcerans*.

We found that the number of BU cases with WHO category 3 lesions decreased significantly and those with category 1 and 2 lesions increased (Table 2). As a result, there was a considerable reduction in patients with joint limitation and treatment with surgery. Furthermore, there was a significant increase in patients confirmed by PCR. These data show that there was an improvement in early detection strategies implemented by the National BU Program in Bénin, of which community health volunteers were integral [34].

## Conclusions

This study confirmed the general decrease in the number of new cases of BU detected in Bénin, as well as in the district of Lalo, from 2006 and 2017. This decrease was observed at the subdistrict and village levels. This study also confirmed the focal character of the geographical distribution of BU. Further research is needed to study the factors associated with this distribution.

## Abbreviations

BU: Buruli Ulcer
CDC: Center of Disease Control and Prevention
CDTUB: Centre de Dépistage et de Traitement de l’Ulcère de Buruli
*MU*: *Mycobacterium ulcerans*
PCR: Polymerase Chain Reaction
WHO: World Health Organization
